# Arsenic-Related Health Risk Assessment of Realgar-Containing NiuHuangJieDu Tablets in Healthy Volunteers *Po* Administration

**DOI:** 10.3389/fphar.2021.761801

**Published:** 2022-01-07

**Authors:** Xiao Wu, Ruoning Yan, Rong Guan, Yi Du, Yuexin Liu, Shanhu Wu, Song Zhu, Min Song, Taijun Hang

**Affiliations:** ^1^ School of Medicine and Holistic Integrative Medicine, Nanjing University of Chinese Medicine, Nanjing, China; ^2^ Key Laboratory of Drug Quality Control and Pharmacovigilance, Ministry of Education, Department of Pharmaceutical Analysis, China Pharmaceutical University, Nanjing, China; ^3^ Department of Pharmacy, First Affiliated Hospital of Zhejiang Chinese Medical University (Zhejiang Provincial Hospital of Traditional Chinese Medicine), Hangzhou, China

**Keywords:** realgar, Niuhuangjiedu tablets, arsenic, health risk assessment, healthy volunteers, hydride generation-atomic fluorescence spectrometry

## Abstract

Realgar, an arsenic-containing traditional Chinese medicine of As_2_S_2_, has significant therapeutic effects for hundreds of years. NiuHuangJieDu tablets (NHJDT) is one of the most commonly prescribed realgar-containing preparations for the treatment of sore throat, swelling, and aching of gums. However, realgar-containing TCMs raise great safety concerns due to the adverse effects reported by arsenic poisoning. In this study, the arsenic-related health risk assessment of NHJDT was conducted in healthy volunteers after single and multiple doses oral administration. Blood, plasma, and urine samples were collected after dosing at predetermined time points or periods. Simple, rapid, and sensitive methods were established for the quantification of total arsenic and arsenic speciation in biological samples. The total arsenic and arsenic speciation were determined by hydride generation-atomic fluorescence spectrometry (HG-AFS) and high-performance liquid chromatography–hydride generation–atomic fluorescence spectrometry (HPLC-HG-AFS), respectively. No significant fluctuation of total arsenic was observed in human blood, and no traces of arsenic speciation were found in human plasma. Dimethylarsenic acid was detected as the predominated arsenic species in human urine after dosing. Therapeutic dose administration of NHJDT was relatively safe in single dose for the limited blood arsenic exposure, but long-term medication may still pose health risks due to the accumulation of arsenics in blood and its extremely slow excretion rate. Therefore, arsenic exposure should be carefully monitored during realgar-containing TCM medication, especially for long-term regimen. The results obtained in this study will provide scientific references for the clinical application of realgar and its-containing TCMs.

## Introduction

Traditional Chinese medicines (TCMs) are effective for many different indications and have been widely used for hundreds of years. Realgar (As_2_S_2_) is prescribed as a mineral medical material since ancient China has shown beneficial effects on carbuncles, psoriasis, malaria, convulsive epilepsy, and parasitic infections (Zhou et al., 2021). Nowadays, realgar-containing TCMs are used more frequently than realgar alone due to the synergistic effects combined with other herbal ingredients (Wu et al., 2018; Liu et al., 2019). There are 38 types of realgar-containing TCMs recorded in the 2020 edition of the Chinese Pharmacopoeia. Among them, NiuHuangJieDu tablets (NHJDT) is the most common and popular realgar-containing preparations for the treatment of sore throat, swelling, and aching of gums (Zhang et al., 2013). It consists of eight ingredients: realgar (6.4%), Huang Qin (*Scutellaria baicalensis* Georgi), Da Huang (*Rheum officinale* Baill.), Jie Geng [*Platycodon grandiflorus* (Jacq.) A. DC*.*], Bing Pian (*Dryobalanops aromatica* C.F.Gaertn*.*), Gan Cao (*Glycyrrhiza glabra L.*), Niu Huang (*Bovis Calculus Artifactus*), and Shi Gao (*Gypsum Fibrosum*).

**Graphical Abstract:**
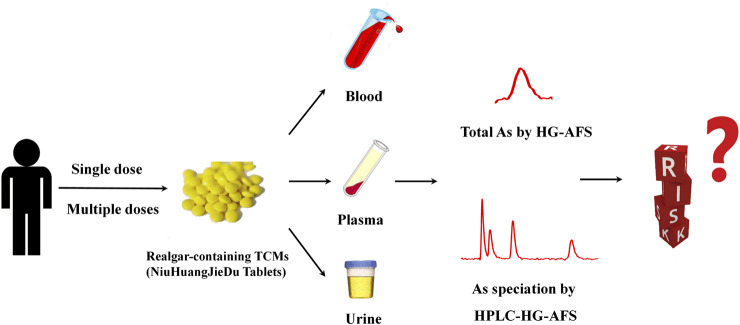


As known to all, arsenic is one of the toxic and carcinogenic elements to induce cancer, cardiovascular disease, peripheral neuropathy, bone marrow depression, and other serious health problems (Kapaj et al., 2006). Realgar mine, located in Shimen County, Hunan Province of China, is the largest realgar deposit in Asia for more than 1500 years. However, thousands of people there are suffering from arseniasis owing to the contaminated water and soil by the waste of arsenic residue in the mining area (Zhu et al., 2010; Tang et al., 2016). Realgar-containing TCMs have also raised great concerns around the world due to the toxicities related with arsenic. Health Sciences Authority (HSA) recalled a TCM ointment for eczema as it had been found with high levels of arsenic (HSA 2015). The Medicines and Healthcare products Regulatory Agency (MHRA) seized a TCM extraction for constipation and dyspepsia which contained overlimit levels of arsenic (57 ppm) (MHRA 2005). The Swedish National Food Agency (SNFA) warned people not to use NHJDT on account of its high levels of arsenic from realgar (MHRA 2013).

However, the water insoluble realgar is not as same as the water soluble toxic inorganic arsenates. Therefore, both realgar and the realgar-containing TCMs should not be deemed as toxic as the equivalent inorganic arsenates. The efficacy of realgar and its-containing TCMs has been generally recognized and widely accepted in China, even though the safety and usage of realgar-containing TCMs still raise questions among the public, and little is known about the risks by the OTC therapeutic usage *in vivo* (Tsoi et al., 2019).

Only our previous studies reported that the NHJDT poses lower risk than realgar in rats through its incorporation with other herbal ingredients (Wu et al., 2018; Wu et al., 2020). However, the affinity of arsenic *in vivo* displayed obvious differences between rats and humans (Lu et al., 2007; Chen et al., 2013). As rat hemoglobin presented a higher affinity for arsenic than human hemoglobin (Lu et al., 2007; Chen et al., 2013), health risk assessment of arsenic in rats is not directly transferable to its safety in humans. Unfortunately, there are still insufficient data for the safety of realgar-containing TCMs in the human body. Hence, health risk assessment of realgar and its-containing TCMs is strongly needed.

To date, several analytical methods have been used for arsenic determination in biological samples, such as hydride generation-atomic fluorescence spectrometry (HG-AFS) and inductively-coupled plasma-mass spectrometry (ICP-MS) (Wegwerth et al., 2021; Liu et al., 2021). HG-AFS is a suitable alternative to ICP-MS in the detection limit, which has the advantages of lower cost, shorter preheating time, and easier operation. A rapid and sensitive high-performance liquid chromatography (HPLC) coupled with the HG-AFS method was also developed for the simultaneous determination of arsenic species in biological samples. The analytical methods of arsenic and its speciation established in this study showed good sensitivity and were appropriate for trace analysis in multiple biological matrices.

In this study, the arsenic-related health risk assessment of realgar-containing NiuHuangJieDu tablets was conducted in healthy Chinese volunteers after a single therapeutic dose of three tablets or multiple doses of three tablets twice a day for seven consecutive days. Total arsenic was determined in human blood and urine by HG-AFS, and the distribution profiles of arsenic speciation in human plasma and urine were also conducted by HPLC-HG-AFS. The arsenic-related health risk of NHJDT was first evaluated in healthy volunteers after single and multiple doses oral administration and will provide useful scientific references for clinical applications and the precautions about the realgar-containing TCMs.

## Materials and Methods

### Materials and Reagents

NHJDT (Batch No.151221090, 0.27 g/tablet) were purchased from Beijing Tong Ren Tang Technologies Co., Ltd (Beijing, China). Arsenic trioxide and the arsenate reference standard were provided by National Institutes for Food and Drug Control (Beijing, China). Dimethylarsenic acid (DMA) was obtained from Huamai Biotechnology Co., Ltd (Beijing, China). Monosodium methanearsonicacid (MMA) was purchased from Laboratories of Dr. Ehrenstorfer GmbH (Augsburg, Germany). Thiourea, ascorbic acid, and potassium borohydride of analytical grade and hydrochloric acid, nitric acid, and sulfuric acid of guaranteed grade were all supplied by Sinopharm Chemical Reagent Co., Ltd (Shanghai, China). Potassium hydroxide, potassium dihydrogen phosphate, and potassium sulfate were provided from Nanjing Chemical Reagent Co., Ltd. (Nanjing, China). HPLC grade methanol was from Tedia Company, Inc (Fairfield, United States). All the other chemicals were of analytical reagent grades. Deionized water was purified on a Millipore Milli Q-Plus purification system (Millipore, MA, United States).

### Apparatus and Analytical Methods

HG-AFS (AF-610D, Rayleigh, Beijing) and HPLC (2695 module, Waters, United States)-HG-AFS were performed for total arsenic and arsenic speciation determination, respectively. The analytical conditions and parameters were consistent with our previously reported studies (Wu et al., 2018; Wu et al., 2020).

### Quality Control of NHJDT

The quality of NHJDT was controlled as recorded in the 2020 edition of Chinese Pharmacopoeia (Supplementary Materials).

The arsenic content of NHJDT was determined by HG-AFS after wet digestion. An aliquot of 20 mg of NHJDT, accurately weighed and placed in a 50 ml Kjeldahl flask with a small glass funnel cover, was mixed with 5 ml HNO_3_-HClO_4_ (4:1). Then, the sample was prepared and determined same, as previously reported (Wu et al., 2018; Wu et al., 2020). Each tablet was determined to contain 17.81 ± 0.79 mg of arsenic.

### Subjects and Setting

Healthy Chinese male or female volunteers aged 18–22 years with a body mass index between 18 and 23 kg/m^2^, without signs of clinical abnormalities from physical examinations and laboratory tests, were enrolled in this study. Exclusion criteria consist of a history of systemic disease, severe allergy or adverse reaction, pregnancy or lactation, and heavy use of alcohol or tobacco. The study design for this OTC drug was approved by the First Affiliated Hospital of Zhejiang Chinese Medical University (Zhejiang Provincial Hospital of Traditional Chinese Medicine) (No. KL-155-01). The informed consents were signed by all the volunteers. All experiments were performed following clinical requirements with medical guidance and monitoring.

The subjects were fasted overnight and received a single therapeutic dose of three tablets or multiple doses of three tablets twice a day for seven consecutive days oral administration of NHJDT.

Nine subjects (4 males and 5 females) were enrolled in the single dose study. Venous whole blood samples of 5 ml were collected into heparinized tubes at 0, 2, 4, 8, 12, 24, and 48 h after dosing, respectively. Plasma samples were prepared from each of the blood samples immediately by centrifugation at 3,000 *g* for 10 min at 4°C. Urine samples were collected at 0–4, 4–8, 8–12, 24, and 48 h after dosing. The volumes of urine samples for each subject were measured and recorded.

Four male subjects were enrolled in the multiple doses study. Venous whole blood samples of 5 ml were collected into heparinized tubes at 0, 2, 4, 8, 12, 24, 48, 96, and 168 h after the final dosing, respectively. The plasma samples were prepared same as those in the single dose study. Urine samples were collected at 0–4, 4–8, 8–12, 24, 48, 96, and 168 h after the final dosing.

The blood, plasma, and urine samples collected were all stored at −80°C until analysis.

### Total Arsenic Determination

An aliquot of 0.5 ml of the whole blood or urine samples were pretreated with a modified fast Kjeldahl digestion, as previously reported (Wu et al., 2018; Wu et al., 2020). Briefly, the calibration standards were prepared with As (III) standards in the concentration range from 0.2–8 ng/ml and 5–100 ng/ml for blood and urine, respectively. Quality control (QC) samples of low (0.5 and 12 ng/ml), medium (3 and 40 ng/ml), and high (6 and 75 ng/ml) levels were prepared by spiking the blank blood or urine with standards separately in the same way (Wu et al., 2018; Wu et al., 2020), respectively. All the test samples were also similarly prepared without the addition of the standard solution and determined by the external standard linear calibration HG-AFS method.

### Arsenic Speciation Determination

An aliquot of 0.2 ml of the plasma or urine samples were prepared as previously reported (Wu et al., 2020). Briefly, the calibration standards for plasma were prepared by spiking the blank human plasma with the working standard solutions in the ranges from 3.125 to 25 ng/ml for As(III), DMA, MMA, and As(V), respectively. QC samples of low (9 ng/ml), medium (10 ng/ml), and high (20 ng/ml) levels were prepared in the same way, respectively. Calibration standards for urine were prepared by spiking the blank urine with all arsenic species standards in the ranges from 3.125 to 250 ng/ml for all the species, respectively. QC samples of low (9 ng/ml), medium (100 ng/ml), and high (200 ng/ml) levels for all the species were prepared in the same way, respectively. All the test samples were prepared similarly without the addition of the mixed species standard solution and determined by the external standard linear calibration HPLC-HG-AFS method.

### Method Validation

The analytical methods established in this study were validated in terms of linearity, limits of detection (LOD), precision, recovery, and stability in accordance with ICH guidelines.

### Pharmacokinetic Analysis

The pharmacokinetic parameters were calculated by the non-compartmental analysis using the WinNonlin program (Pharsight, Palo Alto, CA, United States). Student’s t-test was used for the statistical comparison of pharmacokinetic parameters between single and multiple doses of NHJDT, and the differences were considered to be significant when *p* < 0.05.

## Results and Discussion

The absorbed arsenics tend to have very strong protein binding with the red blood cells and the tissue materials (Lu et al., 2007; Nicolis et al., 2009; Yoshino et al., 2009; Chen et al., 2013), and the elimination was mainly through urine, while the major part of the realgar administered was unattacked and extruded in feces (Tinggi et al., 2016). Therefore, the extent of the possible arsenic-related health risks was evaluated by the total arsenic level determination in human whole blood and urine in this study. Because of the considerably strong protein binding, the arsenics speciation evaluation for the whole blood was practically impossible; therefore, the plasma and urine arsenic species were determined.

### Total Arsenic in Whole Blood

**FIGURE 1:**
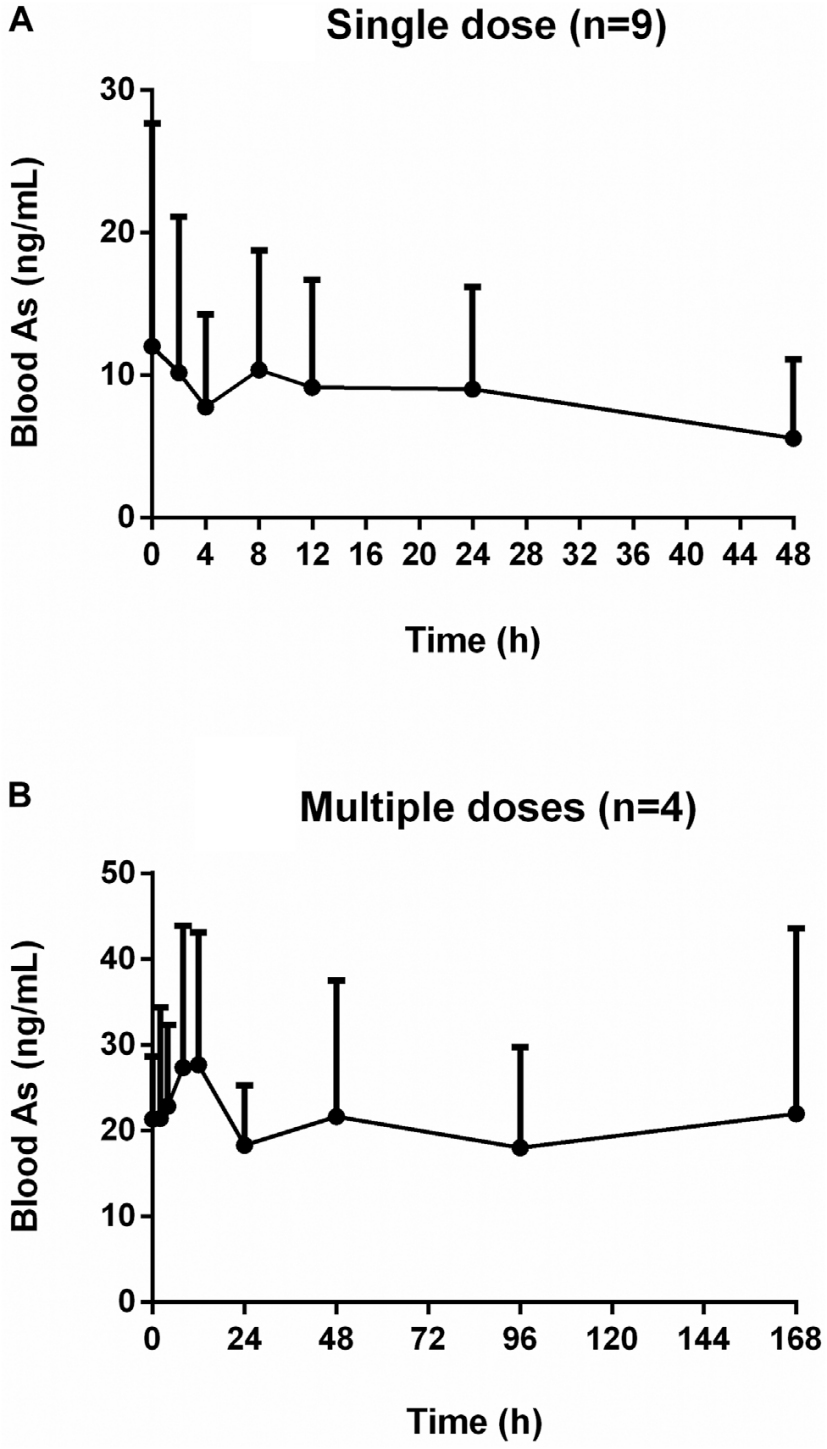
Concentration-time profiles of human blood arsenic after oral administration of NHJDT. **(A)**. single dose (*n* = 9); **(B)**. multiple doses (*n* = 4).

The mean concentration-time profiles of total arsenic in the human blood after the single and multiple doses oral administration of NHJDT are shown in Figure 1. The single-sided error bars in the figure represented the standard deviation of the mean. The pharmacokinetic parameters are presented in Table 1.

**TABLE 1:** Pharmacokinetic parameters of total arsenic in human blood after single and multiple oral doses of NHJDT (Mean ± SD).

Pharmacokinetic parameters	Single dose (*n* = 9)	Multiple doses *(n* = 4)
C_max_ (ng/ml)	20.2 ± 14.2	33.3 ± 13.7^a^
AUC_0-t_ (ng·h/mL)	427 ± 266	3,444 ± 2200^a^
t_1/2_ (h)	27.7 ± 13.2	167.6 ± 23.7^a^
T_max_ (h)	10.5 ± 9.3	7.3 ± 5.0
Accumulation index (AI)	—	20.6 ± 2.8
AUC _multiple doses_/AUC _single dose_	8.1	
C_max multiple doses_/C_max single dose_	1.6	

a Significant difference was found between the single and multiple doses groups (p < 0.05). — : not calculated.

For the single dose study, there was no significant total arsenic level increase in the whole blood after dosing in comparison with the baseline levels about 10 ng/ml. Arsenic exposure varies greatly by geographical location, and blood arsenic levels depend on arsenic ingestion in food and drinking water (Marciniak et al., 2020). Earlier studies reported that blood total arsenic concentrations were 0.82–19.44 ng/ml for unexposed individuals (Lei et al., 2015; Marciniak et al., 2020; Takayama et al., 2021). In our study, the baseline level of arsenic was around 10 ng/ml, which was within the normal range compared to other studies. However, the blood arsenic levels showed an obvious increase after the multiple oral administration. The C_max_ and AUC were 33.3 ± 13.7 ng/ml and 3,444 ± 2200 ng h/mL for the multiple doses administration, respectively. The C_max_ and AUC in the multiple doses were 1.6 and 8.1 times higher than those in the single dose study, respectively. The whole blood total arsenic levels were increased up to about 20 ng/ml at the final dosing time in the multiple doses regimen of NHJDT and were not declined to the baseline levels in the 7-day washing period because of the estimated arsenic’s long elimination half-life of 167.6 ± 23.7 h. And, the accumulation was significant due to the index of 20.6 ± 2.8.

A previous study reported that the therapeutic and toxic concentrations of arsenic in human blood were 2–70 and 50–250 ng/ml, respectively (Schulz and Schmoldt 2003). Because of the extremely low bioavailability of arsenic in realgar and realgar-containing TCMs (Koch et al., 2007; Koch et al., 2011; Lei et al., 2015), blood arsenic levels did not show as much increases as the elevation of the doses. Therefore, NHJDT was relatively safe in single and a short period of multiple doses therapeutic usage with limited blood arsenic exposure. However, it poses obvious health risks of arsenic accumulation when it was multiply administered for long time because of the estimated arsenic’s long elimination half-life and the high accumulation index. These were also clinically proven by a case report that a patient who was ingested with 100 tablets of NHJDT leading to arsenic toxicity though the blood arsenic concentration was found to be only 33 ng/ml (Lam et al., 2018). Thence, great precautions should be taken with NHJDT medication to avoid repeated and long term usage to prevent arsenic accumulation and poisoning.

### Total Arsenic in Urine

**FIGURE 2:**
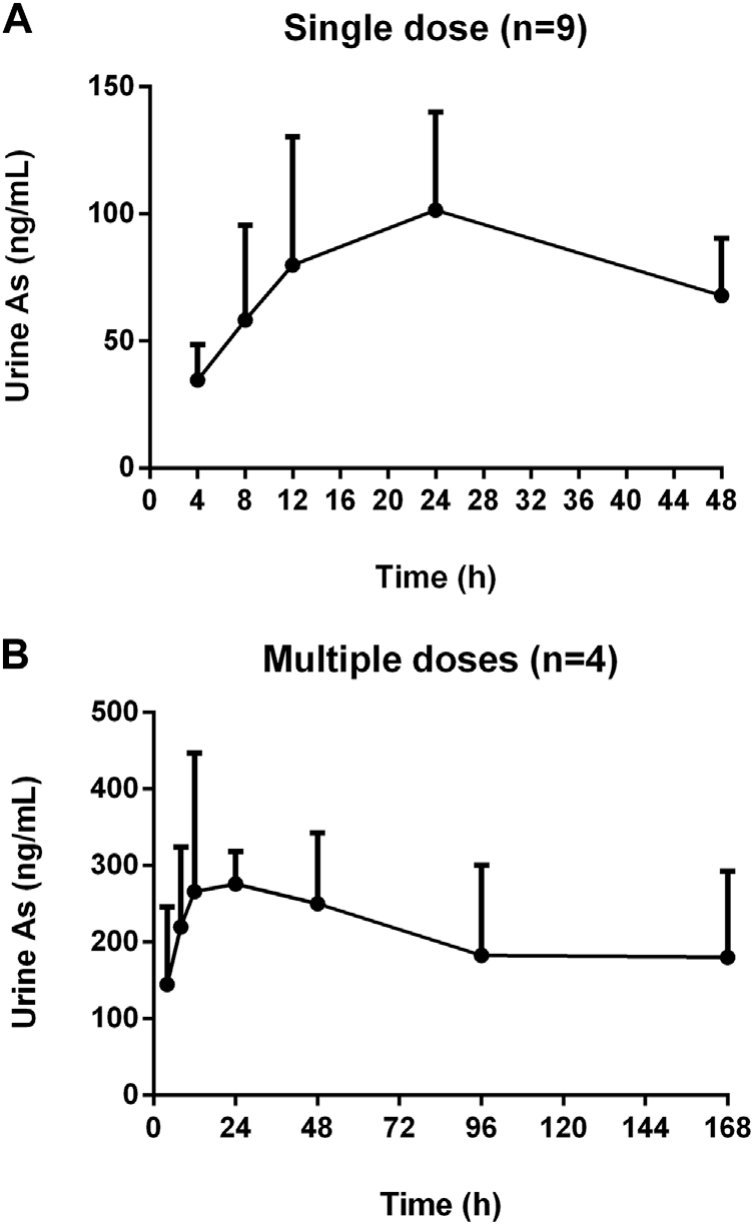
Concentration-time profiles of human urine arsenic after oral administration of NHJDT. **(A)**. single dose (*n* = 9); **(B)**. multiple doses (*n* = 4).

The mean concentration-time profiles of total arsenic in human urine after the single and multiple doses administration of NHJDT are shown in Figure 2, and the corresponding urinary excretion parameters are presented in Table 2. The concentration of total arsenic reached maximum at about 24 h in both the single and multiple groups. The C_max_ with multiple doses was 2.9 times higher than that with the single dose. Urinary total arsenic elimination was considerably slow because of the non-obvious decrease of the urine concentration after 48 and 168 h with the single and multiple doses, respectively. Therefore, the total arsenic concentration in urine also demonstrated the significant arsenic exposure and accumulation and the corresponding possible health risks with a long-time regimen of NHJDT. This was also confirmed by a case report from an arsenic poisoning patient that the urinary arsenic exposure presented 17 times higher than that of the blood (Lam et al., 2018). Therefore, the urinary arsenic exposure is a more sensitive indicator of the potential health risks of realgar and its-containing TCMs.

**TABLE 2:** Urinary excretion parameters of total arsenic in human urine after single and multiple oral doses of NHJDT (Mean ± SD).

Urinary excretion parameters	Single dose	Multiple doses
C _max_ (ng/ml)	111 ± 34	327 ± 65^a^
AUC _0-t_ (ng·h/mL)	3,597 ± 1139	35,651 ± 14,344^a^
T_max_ (h)	25.3 ± 14.0	15.0 ± 6.0^a^

a Significant difference was found between the single and multiple doses groups (p< 0.05).

### Arsenic Speciation in Plasma and Urine

**FIGURE 3:**
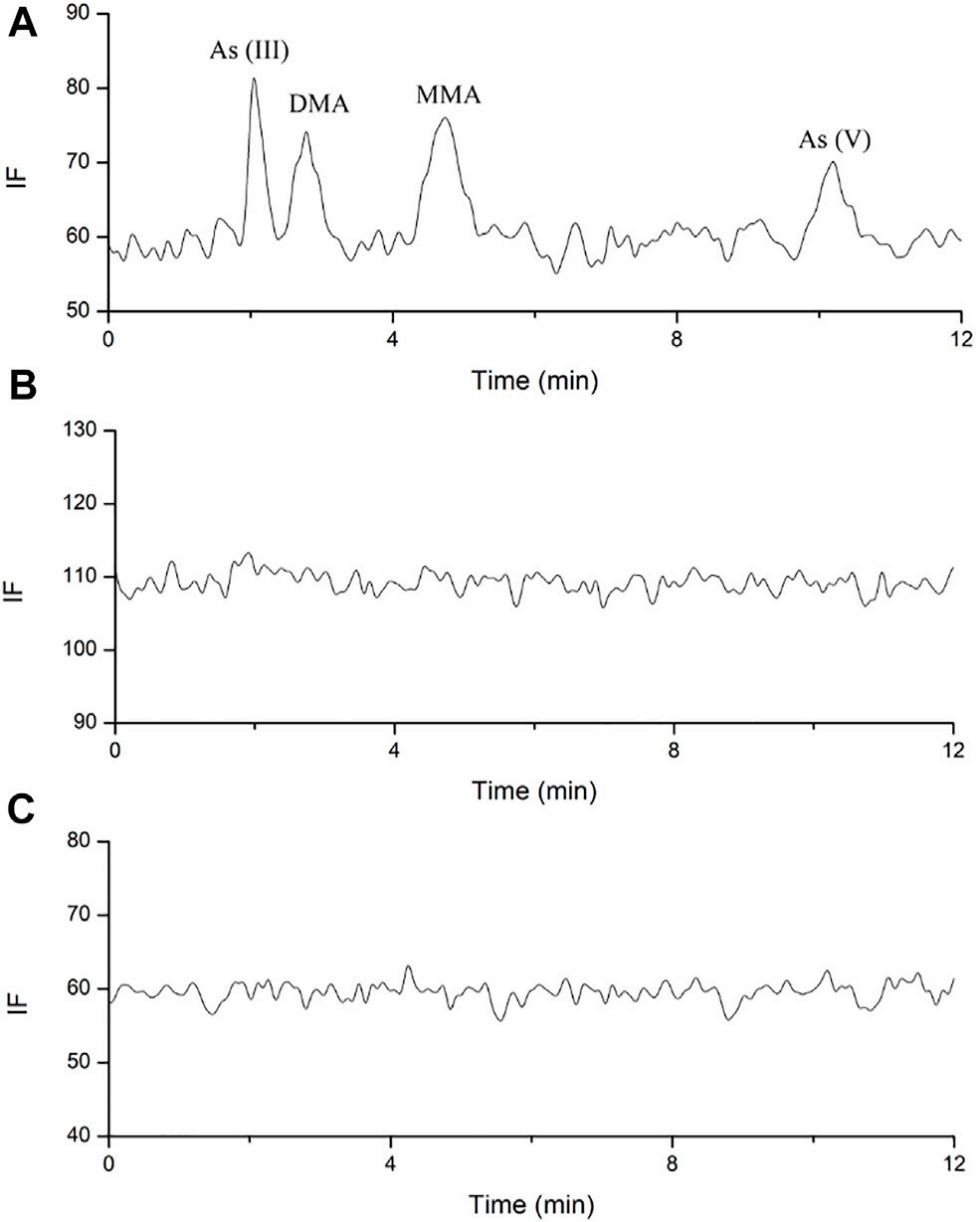
Representative HPLC-HG-AFS chromatograms of arsenic species in human plasma. **(A)**. blank spiked with arsenic species of 3.125 ng; **(B)**. sample after single oral administration; **(C)**. sample after multiple oral administration.

**FIGURE 4:**
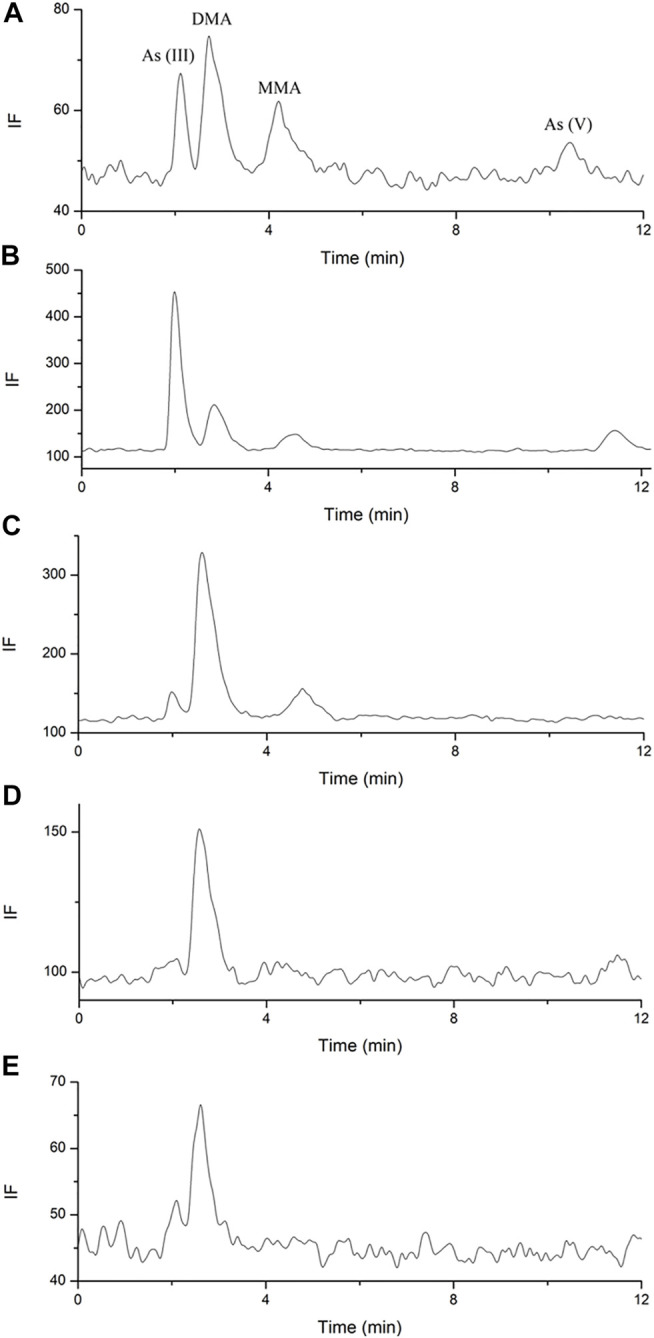
Representative arsenics urine species chromatograms. **(A)**. blank spiked with arsenic species each of 3.125 ng; **(B)**. sample of 4–8 h after single dose; **(C)**. sample of 48 h after single dose; **(D)**. sample of 10 days after single dose; **(E)**. sample of 38 days after multiple doses.

**FIGURE 5:**
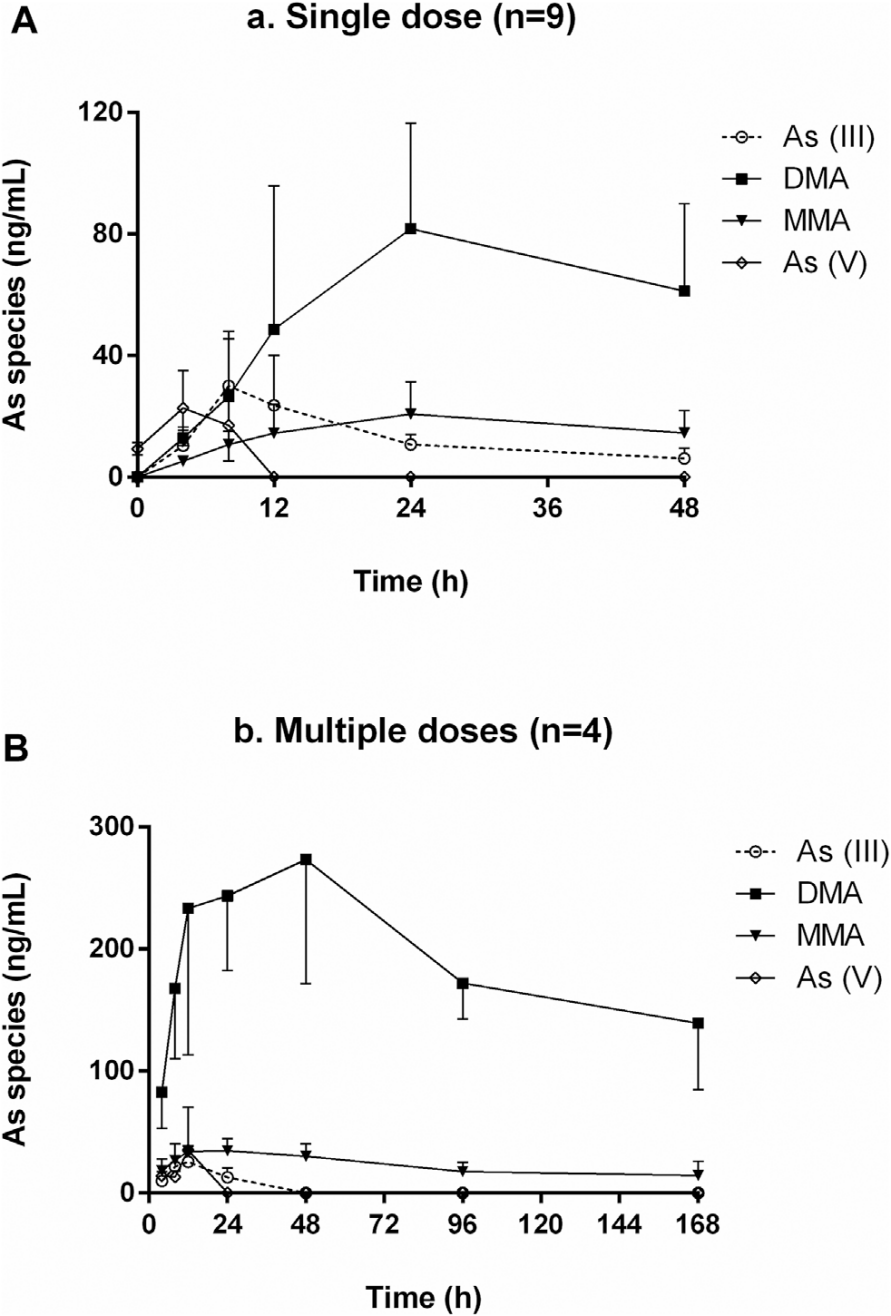
Concentration-time curves of As (III), DMA, MMA, and As (V) in human urine after oral administration of NHJDT. **(A)**. single dose (*n* = 9); **(B)**. multiple doses (*n* = 4).

**FIGURE 6:**
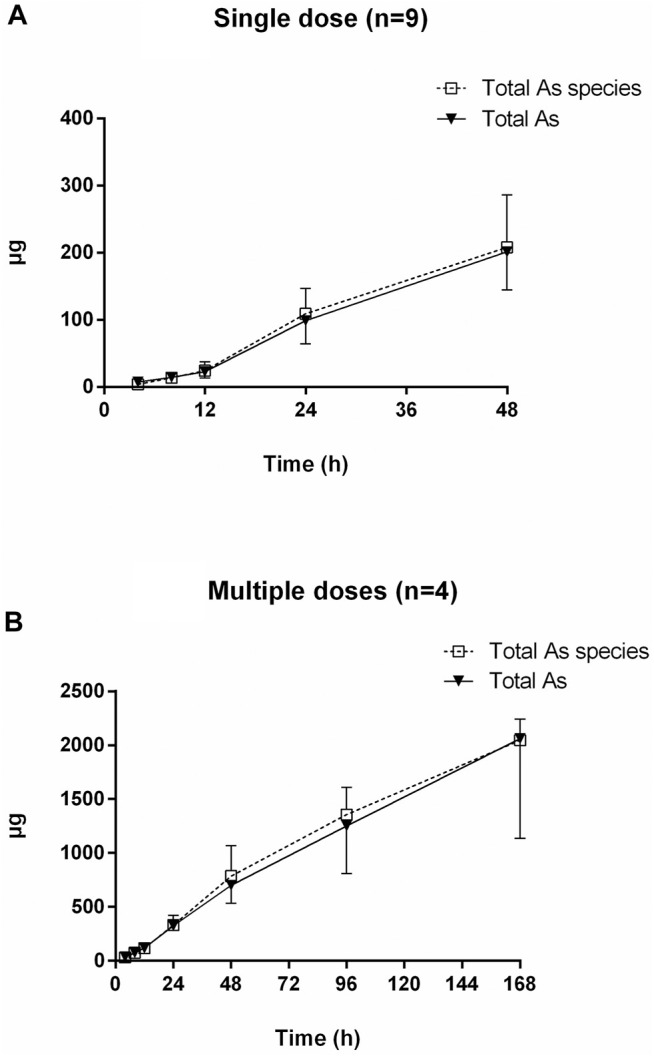
Cumulative excretion of total arsenic and total arsenic species after oral administration of NHJDT. **(A)**. single dose (*n* = 9); **(B)**. multiple doses (*n* = 4).

The plasma and urine arsenic speciation profiles found by the validated HPLC-AFS method are shown in Figures 3–6. As(III), DMA, MMA, and As(V) achieved a good separation under the optimized chromatographic conditions.

No arsenic species was found above the LODs (1 ng/ml) in the plasma for all the components under investigation after both the single and multiple doses oral administration (Figure 3). As reported in a previous study, DMA, MMA, and inorganic arsenics were detected in the plasma of acute promyelocytic leukemia (APL) patients after daily intravenous administration of arsenic trioxide at 0.16 mg/kg (Guo et al., 2021). In our study, no traces of arsenic speciation were found in the human plasma because the absorption of arsenic was considerably low after oral administration of NHJDT, and the limited amount of arsenic in the blood was substantially bound to red blood cells (Lu et al., 2007; Nicolis et al., 2009; Yoshino et al., 2009; Chen et al., 2013). High affinity of arsenic with human hemoglobin was responsible for the accumulation of arsenic in the blood of APL patients (Guo et al., 2021), which was consistent with our study. Therefore, the whole blood total arsenic level is confirmed to be a more reliable indicator than that of plasma for arsenic-related health risk assessment.

HPLC-HG-AFS chromatograms of arsenic speciation in human urine after the single and multiple doses of NHJDT are shown in Figures 4, 5, respectively. DMA was detected as the predominate species in human urine after dosing, and a small amount of MMA, As (III), and As (V) was also detected. In agreement with previous reports, DMA was the predominant species in urine, and it increased to the maximum within 15–25 h and then decreased after single ingestion of arsenic-containing substances (Acuña et al., 2014; Hulle et al., 2004). As presented in Figure 5, as the concentration of DMA and MMA had a significant increase in urine for the first 48 h post administration, both As (III) and As (V) had only very short duration of appearance within 24 h. Previous studies have documented that arsenic methyltransferase involved in arsenic metabolism, and the methylated metabolites of MMA and DMA were excreted in urine (Aposhian et al., 2004; Nicolis et al., 2009; Watanabe and Hirano, 2013; Khairul et al., 2017). Arsenic methylation observed in human urine of this study was highly in consistence with these previously reports. Because of the lower toxicity of the organic arsenics than inorganic arsenics (Zhang et al., 2013; Zhou et al., 2021), the methylated metabolites detected in human urine poses a lower risk in human body.

The urinary cumulative excretion of total arsenic and total arsenic species is shown in Figure 6. No significant difference was observed between the content of total arsenic and total arsenic species in human urine, indicating that all the arsenic species were totally detected by the established HPLC-HG-AFS method. The 24 and 48 h cumulative amount of total arsenic excreted was 326 ± 37 and 702 ± 168 μg for the multiple doses, which were 3.3–3.5 times higher than the single dose data of 99 ± 34 and 202 ± 57 μg, respectively. The total urinary cumulative excretion rate of arsenic was 0.38 and 0.28% in single and multiple doses, respectively. The extremely low urinary arsenic excretion illustrated that urinary excretion is not the dominant excretion pathway of arsenics and most of the arsenics should be the unattacked realgar and excreted in the feces, which was verified in a previous study (Yi et al., 2020).

The results revealed that urinary arsenic was not easily eliminated. Therefore, the urine samples were further collected from the subjects at 10 and 38 days post the single and multiple doses administration, respectively. As shown in Figures 4D,E, DMA can still be detected in human urine 10 days after the single dose or 38 days after the multiple doses. The slow elimination of arsenics from the human body is clinically once more proven because of the strong protein and tissue binding (Guo et al., 2021). Thence, precautions should be taken to avoid excessive or long-term medication of realgar-containing TCMs to prevent high levels of arsenic exposure. More detailed instructions of arsenic-related health risks should be labeled in realgar-containing preparations.

A therapeutic dose of NHJDT was relatively safe because of the limited total blood arsenic and the absence of arsenic species in human plasma. Urinary total arsenic elimination was considerably slow, and DMA was the predominant species in urine after oral administration of NHJDT. The urinary arsenic exposure is a more sensitive indicator than blood arsenic to assess the health risk of realgar and its-containing TCMs.

### Method Validation

The linear calibrations in this study were found with a coefficient of *R* ^2^ to be greater than 0.999. The LODs of total arsenic and arsenic speciation were 0.02 and 1 ng/ml by HG-AFS and HPLC-HG-AFS, respectively. The precisions and accuracies of QC samples were all within 15% and 85%–115%, respectively.

The recoveries were determined by addition of a known amount of arsenic to the known content of the test sample. The overall recoveries were found to be 85–115% with the RSDs less than 15%.

As all the arsenics in biological samples were all converted to As(V) by digestion regardless of storage temperature and time and freeze–thaw cycles, thence the stability of total arsenic was only investigated with the finally reduced digestion solutions prepared by QC samples stored at room temperature for 48 h with an RSD less than 5%. The arsenic species were found to be stable when stored at room temperature for 6 h, after three freeze-thaw cycles, and at 4°C in the autosampler for 12 h.

## Conclusion

Arsenic-related health risk assessment of realgar-containing NHJDT was conducted in healthy volunteers after single and multiple doses oral administration. No significant fluctuation of total arsenic was observed in the human blood, and no traces of arsenic speciation were found in human plasma. DMA was detected as the predominated arsenic species in human urine after dosing. Therapeutic dose administration of NHJDT was relatively safe in single dose with a limited blood arsenic exposure, but long-term medication will certainly pose health risks due to the accumulation of arsenics in the human body and its extremely slow excretion rate. Thence, attention should be paid to realgar and its-containing TCMs to prevent excessive arsenic exposure. The study first evaluated the profiles of arsenic in the human body after oral administration of NHJDT and will be helpful for the clinical application of realgar-containing TCMs.

## Data Availability

The raw data supporting the conclusion of this article will be made available by the authors, without undue reservation.
