# Supplementary material for: Arsenic-Related Health Risk Assessment of Realgar-Containing NiuHuangJieDu Tablets in Healthy Volunteers Po Administration
Source: Front Pharmacol. 2022 Jan 7;12:761801. doi: 10.3389/fphar.2021.761801 (PMC8776706; doi:10.3389/fphar.2021.761801)
Supplement: Supplementary file 1 [file DataSheet1.docx]

**Supplementary Materials and methods**

1.1. Preparation of Niuhuangjiedu Tablet (NHJDT)

NHJDT (Batch No.151221090, 0.27 g/tablet) were purchased from Beijing Tongrentang Technologies CO., Ltd. (Beijing, China). It is officially listed in the current Chinese Pharmacopeia with eight herbal components including realgar, Da Huang (*Rheum officinale Baill.*), Huang Qin (*Scutellaria baicalensis Georgi*), Jie Geng (*Platycodon grandiflorus (Jacq.) A.DC.*), Bing Pian (*Dryobalanops aromatica C.F.Gaertn.*), Gan Cao (*Glycyrrhiza glabra L.*), Niu Huang (*Bovis Calculus Artifactus*),and Shi Gao (*Gypsum Fibrosum*). NHJDT was prepared by decoting *Scutellaria baicalensis Georgi* (150 g)*, Gypsum Fibrosum* (200 g)*, Platycodon grandiflorus (Jacq.) A.DC.* (100 g) *and Glycyrrhiza glabra L.* (50 g) with water for 2 h for twice. The filter was merged and concentrated, and the extractum was mixed with *Rheum officinale Baill.* (200 g) and realgar (50 g) to form granules*.* Then mix with *Bovis Calculus* *Artifactus* (5 g) and *Dryobalanops aromatica C.F.Gaertn.* (25 g) fine powder, dried and pressed into 1500 pieces, sugar coated or film coated.

Table S1 The formula of Niuhuangjiedu Tablet (NHJDT)

| Herbal name | Chinese name | Weight (g) |
| --- | --- | --- |
| *Rheum officinale Baill.* | Da Huang | 200 |
| *Gypsum Fibrosum* | Shi Gao | 200 |
| *Scutellaria baicalensis Georgi* | Huang Qin | 150 |
| *Platycodon grandiflorus (Jacq.) A.DC.* | Jie Geng | 100 |
| *Glycyrrhiza glabra L.* | Gan Cao | 50 |
| *Realgar* | Xiong Huang | 50 |
| *Dryobalanops aromatica C.F.Gaertn.* | Bing Pian | 25 |
| *Bovis Calculus* *Artifactus* | Niu Huang | 5 |

1.2. HPLC analysis

To guarantee the quality of the NHJDT, HPLC was employed to detect baicalin, which is the typical active component in the formula. According to the latest Chinese pharmacopoeia, the content of baicalin must not be less than 3.0 mg for each tablet. The chromatographic conditions for baicalin content determination were set as follows:

Mobile phase: Methanol-water-phosphoric acid (45:55:0.2)

Wavelength: 315 nm

Flow rate: 1.0 mL/min

Column temperature：35°C

Column：Agilent XDB-C18 (4.6×250 mm, 5 μm) (Agilent Technologies, Inc., USA).

Instrument： High Performance Liquid Chromatography (2695 module, Waters, USA)

Injection volume：10 μL

Sample preparation:

Took finely powdered 0.6 g of NHJDT, accurately weighed, and placed into a volumetric flask. Then 30 mL of 70% ethanol was added, and extracted for 20 min with ultrasonic. The filtrate was transferred into a 100 mL volumetric flask with 70% ethanol. 2 mL of the above solution was transferred accurately, and dilute to 10 mL with 70% ethanol.


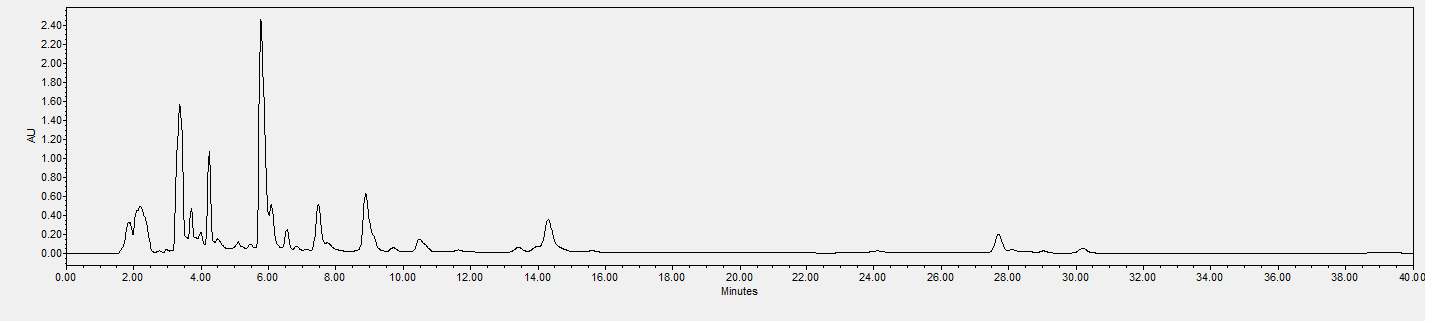


Fig. S1 Representative HPLC chromatogram of NHJDT

Table S2. The HPLC retention times of active components of NHJDT.

| Compound | Retention time (min) |
| --- | --- |
| Baicalin | 5.776 |
| Wogonoside | 8.887 |
| Wogonin | 27.705 |
| Chrysin | 28.572 |
| Oroxylin A | 30.198 |

Each tablet contained 4.19 mg of baicalin, which conformed to the standard of NHJDT recorded in Chinese Pharmacopeia.
